# Supplementary material for: Blocking of P2X7r Reduces Mitochondrial Stress Induced by Alcohol and Electronic Cigarette Exposure in Brain Microvascular Endothelial Cells
Source: Antioxidants (Basel). 2022 Jul 6;11(7):1328. doi: 10.3390/antiox11071328 (PMC9311929; doi:10.3390/antiox11071328)
Supplement: Supplementary file 1 [file antioxidants-11-01328-s001.zip › antioxidants-1763685-supplementary.pdf]

<u>**Supplementary Data:**</u>

Dose response study for compound A804598 (A80) by mito-stress test:

Method:

hBMVECs (30,000cells/well) plated in seahorse XF96 microplate were allowed to attach overnight. Next day cells were treated with 5µM, 10 µM and 20 µM of the A80 compound. After overnight incubation, cytotoxic levels of A80 compound were checked by measuring mito-stress levels in hBMVECs. Simultaneously, we tested the effective concentration A80 compound against a potent secondary metabolite of ethanol, that is acetaldehyde (ALD). For seahorse working protocol please refer our original manuscript.

Result:

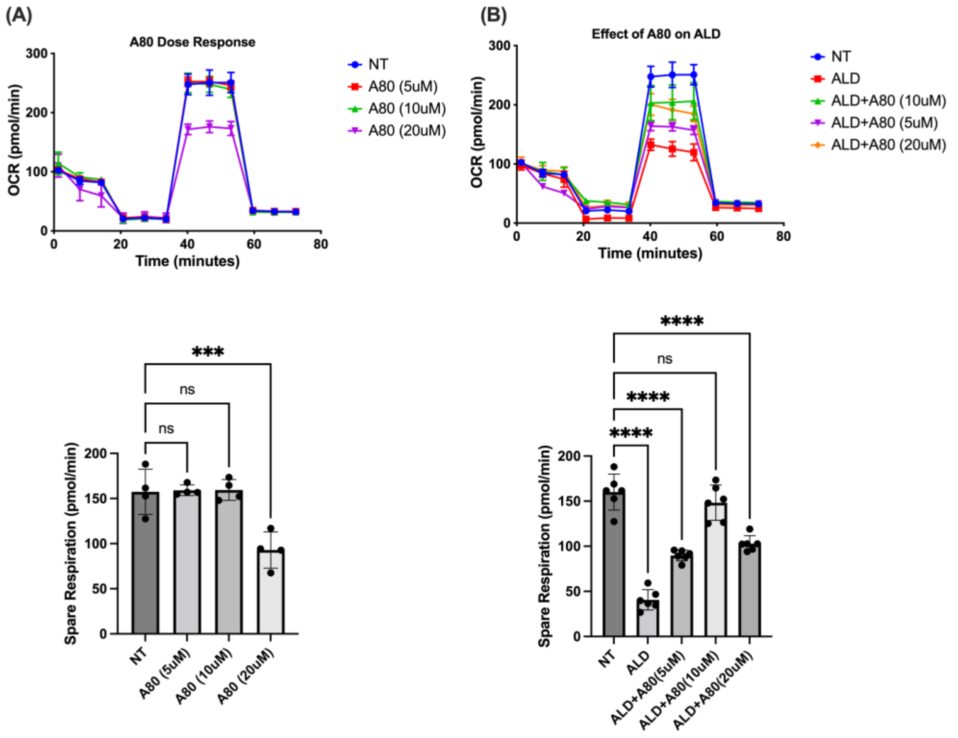

Figure S1. (A) After overnight incubation, lower concentrations of A80 (5µM and 10µM) were found to be non-cytotoxic and had no effect on mitochondrial function. However, increase in concentration to 20µM seemed to be toxic to hBMVECs and significantly reduced the mitochondrial spare respiration levels. **(B)** While testing effective concentration of A80 against ALD, our Seahorse data provided a solid evidence that A80 compound alleviated mitochondrial stress across all the concentrations we tested. However, at a lower concentration (5µM) the effect of A80 compound on spare respiration levels did not reach statistical significance and higher concentration (20µM) A80 seemed to be cytotoxic. Within this dose range, only 10µM of A80 was found to be an effective concentration that significantly improved the spare respiration with minimal cytotoxic effects (n=4-6). We normalized the Seahorse data to untreated control cells and One-Way ANOVA was used for statistical analysis, marked as ***$P \leq 0.001$, ****$P \leq 0.0001$ and ns (not significant).
